# Transcatheter aortic valve replacement failure: surgical valve explantation after more than a decade

**DOI:** 10.1093/icvts/ivae177

**Published:** 2024-10-26

**Authors:** Go Yamashita, Shingo Hirao, Tatsuhiko Komiya

**Affiliations:** Department of Cardiovascular Surgery, Kurashiki Central Hospital, Kurashiki, Okayama, Japan; Department of Cardiovascular Surgery, Kurashiki Central Hospital, Kurashiki, Okayama, Japan; Department of Cardiovascular Surgery, Kurashiki Central Hospital, Kurashiki, Okayama, Japan

**Keywords:** Transcatheter aortic valve replacement, Surgical valve explantation, Aortic valve insufficiency

## Abstract

Transcatheter aortic valve replacement (TAVR) has become an established alternative to surgical aortic valve replacement for severe aortic stenosis. However, the long-term outcomes and need for surgical reintervention following TAVR remain uncertain. This case report describes a 76-year-old woman who underwent surgical explantation of a SAPIEN-XT valve more than a decade after initial TAVR implantation due to late valve failure. The patient presented with severe aortic insufficiency and heart failure symptoms. Surgical intervention involved concomitant ascending aortic replacement, tricuspid annuloplasty and coronary artery bypass grafting. The TAVR valve was successfully explanted using careful blunt dissection to avoid annulus damage. Postoperative recovery was uneventful, with the patient discharged after 4 weeks. This case highlights the potential need for long-term surgical management of patients after TAVR and emphasizes the importance of surgical preparedness as TAVR indications expand. It also provides valuable insights for surgeons encountering similar cases of late TAVR failure requiring explantation.

## INTRODUCTION

Transcatheter aortic valve replacement (TAVR) is an established alternative to surgical aortic valve replacement (SAVR) for severe aortic stenosis. The number of TAVRs performed has increased rapidly, and its indications have expanded to include younger populations. However, the long-term outcomes and surgical reintervention required for TAVR valves after extended periods remain unknown.

This report discusses the case of a 76-year-old woman who underwent TAVR with a SAPIEN-XT valve over 10 years before the presentation and was admitted for surgical explantation due to late valve failure. This case highlights the importance of surgical preparedness as TAVR indications expand.

## CASE DESCRIPTION

A 76-year-old woman with severe aortic valve insufficiency (AI) who had undergone TAVR with a balloon-expandable 23-mm SAPIEN-XT valve 10 years before presentation was transferred to our hospital for surgical intervention due to heart failure and dyspnoea at rest. TAVR was initially chosen due to multiple comorbidities, including a porcelain aorta. Assessment using the Society of Thoracic Surgeons predicted the mortality risk for the initial TAVR in this case to be 3.0%. Post-TAVR echocardiography revealed mild AI caused by perivalvular leakage. One month before the transfer, echocardiography showed severe AI due to deteriorating transvalvular leakage and moderate aortic stenosis. Given the complex nature of the valve dysfunction and the patient’s overall condition, elective SAVR was chosen over redo-TAVR or transcatheter perivalvular leak closure. Considering the porcelain aorta, tricuspid valve regurgitation and coronary artery stenosis, the patient underwent concomitant ascending aortic replacement, tricuspid annuloplasty and coronary artery bypass grafting.

After cardiac arrest, distal anastomosis was performed using a 26-mm single-branched polyester woven graft (J Graft SHIELD NEO; Junken Medical Co., Ltd, Tokyo, Japan) under mild hypothermic circulatory arrest with selective cerebral perfusion.

**Figure 1:**
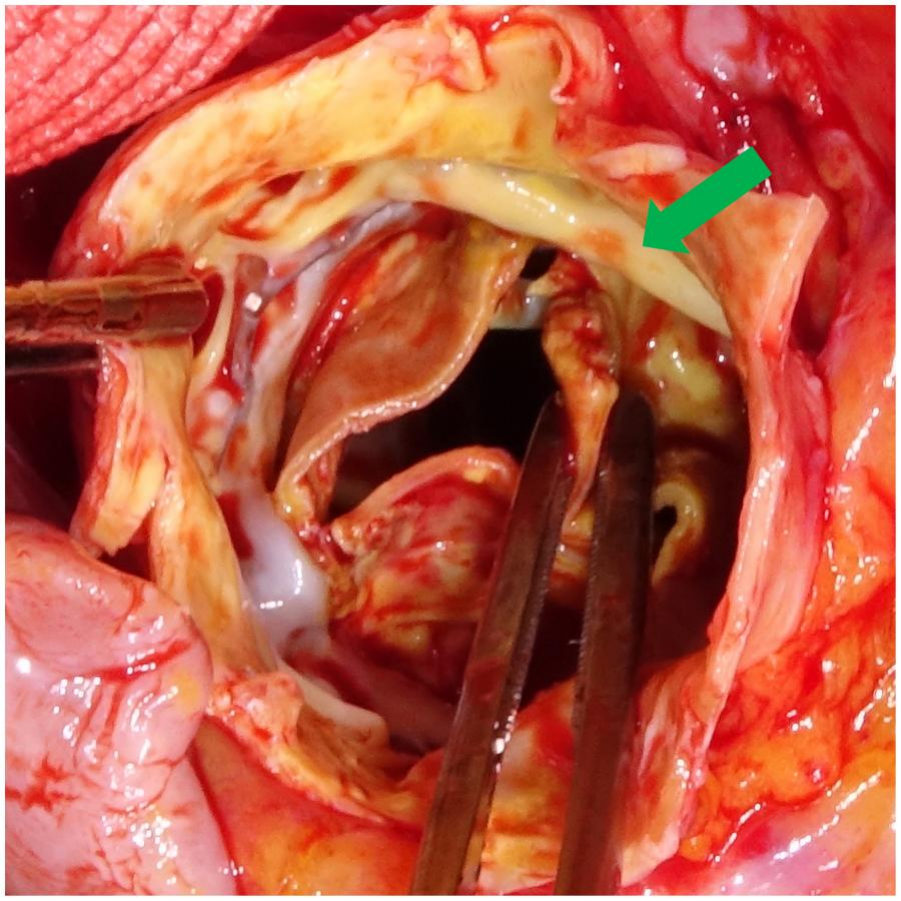
SAPIEN-XT valve is adherent to the endothelium, with a visible tear on the right coronary cusp (green arrow).

**Figure 2:**
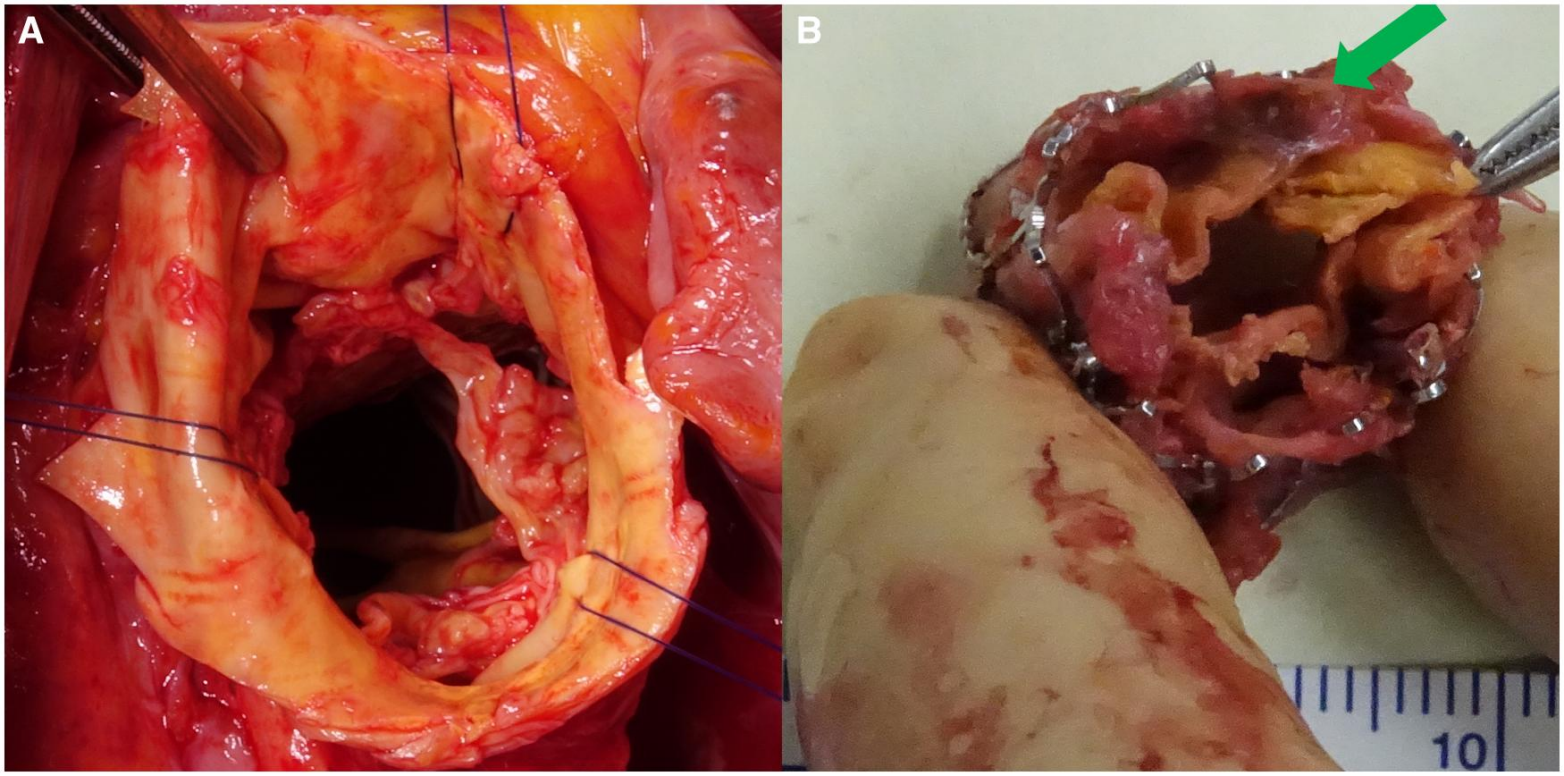
(**A**) Intraoperative aortic valve after explantation. (**B**) Explanted SAPIEN-XT valve with a tear near the commissure left of the right coronary cusp (green arrow).

Upon restarting circulation, we identified that the distal rim of the SAPIEN-XT valve stent was exposed, and the stent and proximal portion of the valve were strongly adherent to the endothelium at the level of the valve skirt (Fig. 1). We used the Kocher clamp manoeuvre to assist in transcatheter valve removal, carefully explanting the valve using blunt dissection to avoid annulus damage (Fig. 2A). The TAVR was torn near the commissure on the left side of the right coronary cusp (Fig. 2B). The native cusps were excised and replaced with a 21-mm Magna Ease (Edwards Lifesciences, Irvine, CA, USA) valve (Video 1). Subsequently, tricuspid annuloplasty and coronary artery bypass grafting were performed. The postoperative course was uneventful, and the patient was discharged after 4 weeks. At writing, after 2.5 years of uneventful outpatient follow-up, echocardiography showed no residual paravalvular leak of the implanted valve.

Ethics approval is not required for case reports at our institution. The patient provided written informed consent for the publication of this article.

## DISCUSSION

The indications for TAVR have rapidly expanded, initially including only high-risk cases and now including cases with intermediate or low risk [1]. As more TAVR procedures are performed, the annual incidence of explantation has increased. Fukuhara *et al.* [2] reported a mortality rate of 14.2% after explantation, particularly in native TAVR. In the scarce reports on TAVR explantation, the median time from initial TAVR to explantation ranged from 1.0 to 3.0 years, indicating that only relatively early explantations have been reported [2, 3]. With the Notion trial reporting 10-year outcomes for TAVR, the number of TAVR explantations is expected to increase [4]. In the present case, TAVR valve explantation was successful more than 10.4 years after the initial implantation.

Compared with other TAVR valves, the SAPIEN-XT valve is short, making it easier to dissect and remove without damaging the surrounding native valve tissue, although explantation of other valve types may present different challenges. In cases of isolated structural valve deterioration, redo-TAVR is often preferred [5]. However, in complex cases with a combination of stenosis and regurgitation, including perivalvular leakage, surgical explantation may be best. To the best of our knowledge, no case of TAVR valve explantation ≥10 years after implantation has been reported. As the indications for TAVR expand, an exponential increase in TAVR explantations is expected. In this context, SAVR is expected to become more common in cases in which explantation is performed >10 years after TAVR. We believe that this report will assist surgeons in encountering similar cases. Regarding long-term valve degeneration (>10 years), although data are still limited, factors such as persistent haemodynamic stress, suboptimal valve positioning and individual patient characteristics may play a crucial role. The present case, with its late presentation of failure, underscores the need for vigilant long-term follow-up and further research on long-term TAVR outcomes.

## Data Availability

The data from this study will be shared on reasonable request to the corresponding author.
